# Health inequalities after austerity in Greece

**DOI:** 10.1186/s12939-016-0374-0

**Published:** 2016-05-31

**Authors:** Marina Karanikolos, Alexander Kentikelenis

**Affiliations:** European Observatory on Health Systems and Policies, London School of Hygiene and Tropical Medicine, London, UK; Department of Sociology, University of Oxford, Oxford, UK

**Keywords:** Greece, Economic crisis, Access to care, Unmet need, Inequality, Austerity

## Abstract

Since the beginning of economic crisis, Greece has been experiencing unprecedented levels of unemployment and profound cuts to public budgets. Health and welfare sectors were subject to severe austerity measures, which have endangered provision of as well as access to services, potentially widening health inequality gap. European Union Statistics on Income and Living Conditions data show that the proportion of individuals on low incomes reporting unmet medical need due to cost doubled from 7 % in 2008 to 13.9 % in 2013, while the relative gap in access to care between the richest and poorest population groups increased almost ten-fold. In addition, austerity cuts have affected other vulnerable groups, such as undocumented migrants and injecting drug users. Steps have been taken in attempt to mitigate the impact of the austerity, however addressing the growing health inequality gap will require persistent effort of the country’s leadership for years to come.

## Background

Entering in the 9th year of economic crisis in 2016, Greece has witnessed a 29 % drop in its gross domestic product (GDP) between 2008 and 2014, while, as of 2014, the unemployment rate reached 26.5 % and long-term unemployment 19.5 %. The crisis—and the associated policy response designed by Greece’s international creditors—resulted in sharp reductions in public expenditures amounting to a 36 % drop between 2009 and 2014 [1].

## Main text

A range of developments in the country have endangered the provision of health and welfare services as well as people’s ability to access them, which—in turn—can have adverse impacts on health equity. But have some population groups been affected more than others?

First, as in Greece access to health care is largely determined by employment, people without jobs as well as their family members (estimated to exceed 2 million) have been left without comprehensive health coverage [2]. Second, the health sector itself has been the target of a persistent revenue-raising and cost-cutting drive, with low-income households being disproportionately affected. Recent reforms have included the introduction of user fees to access hospital services, increased co-payments for pharmaceuticals, discontinuation of programmes for vulnerable populations, and long waiting lists for access to health services [3]. Finally, the overall economic climate as a result of the crisis—high unemployment, fear of job loss, and loss of income—can also have an impact on health inequalities, as they are link ed to higher general mortality [4, 5], deteriorating mental and physical health [6], and higher exposure to determinants of ill-heath [7].

**Fig. 1:**
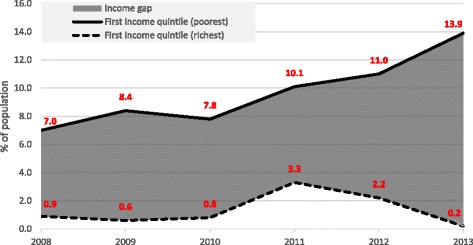
Inequalities in unmet need medical due to cost by income level, Greece, 2008-2013

The latest available data from representative population survey European Union Statistics on Income and Living Conditions (EU-SILC) reveals that Greece’s worsening economic conditions have undermined access to health care services, particularly for those most vulnerable. The proportion of individuals on low incomes reporting unmet medical need due to cost doubled from 7 % in 2008 to 13.9 % in 2013 (Fig. 1). At the same time, self-reported unmet need in the richest population quintile is at the lowest level since the beginning of the crisis, leading to the increase of the relative gap between the richest and poorest population groups to almost ten-fold between 2008 and 2013 [1].

In addition, the most vulnerable groups who frequently are left invisible to official statistics and population surveys are at the forefront of feeling the consequences of the austerity. A study focusing on undocumented migrants demonstrated that 62 % had unmet health need, while 53 % had major difficulty in accessing health services, with key barriers being the cost and long waiting lists [8]. Access to health services, including emergency and inpatient treatment, medical examinations and mental health care deteriorated for homeless people during the recession [9]. Cuts to already modest disease prevention programmes have led to HIV outbreak among the injecting drug users in 2011-13, while incidence of tuberculosis more than doubled in this population in 2013 compared to 2012 [3]. Provision of services for these vulnerable groups rely largely on charities, which now also have to cope with increasing demand from impoverished general population, as user charges for healthcare services and pharmaceuticals affect large proportion of households with low income.

Since the issue of the impact of crisis and austerity on health and access to care in Greece was raised a few years ago [10], a number of actions were taken. For instance, a health voucher scheme intended to cover 230,000 people with basic services only covered 10 % of these in the first 17 months since its introduction in 2013. The legislative initiative from June 2014 which enabled access to primary and inpatient care and to pharmaceuticals for the uninsured in practice was hampered by rigid bureaucratic means-testing procedures for eligible groups and lack of information for healthcare providers [11]. These measures to mitigate the impact of crisis and austerity on the public health in Greece have come too little and too late. The growing health inequality gap suggests that the Greek welfare state—reeling under the pressure of exhaustive austerity—has failed to live up to its promise of universal health coverage.

## Conclusions

Chronic as well as recent ailments of Greece’s health system continue to affect its capacity to adequately shelter the social groups most affected by the crisis. Recent political turmoil in 2015—including two national elections, a referendum, a Eurozone membership crisis, and the refugee crisis—have drawn attention away from the pressing challenges for the country’s health system. Beginning to address the growing health inequality gap will require the coordinated and persistent effort of the country’s leadership over the years to come.

## Abbreviations

EU-SILC, European Union Statistics on Income and Living Conditions; GDP, gross domestic product.
